# Neurosyphilis with stroke-like manifestations: A case report

**DOI:** 10.1097/MD.0000000000042294

**Published:** 2025-04-25

**Authors:** Ling Zeng, Bin Wu, Yushi Zhong

**Affiliations:** a Department of Neurology, Hunan University of Medicine General Hospital, Huaihua, PR China.

**Keywords:** case report, neurosyphilis, stroke

## Abstract

**Rationale:**

Neurosyphilis is a relatively common condition that can have stroke-like episodes and is usually seen as an ischemic lesion on magnetic resource imaging (MRI). However, we found a case of diffusion-weighted imaging (DWI)-negative neurosyphilis with stroke-like episodes.

**Patient concerns:**

After hospitalization, MRI of the head was perfected and no new cerebral infarct lesions were seen, and DWI was negative. However, the patient’s left limb weakness was significantly aggravated, and serological examination suggested a positive syphilis test.

**Diagnoses:**

The patient had a sudden onset of weakness in the left limb. Computed tomography (CT) and MRI brain scans were performed. A CT scan of the head showed no significant abnormalities. MRI of the head showed multiple flaky abnormal signal shadows in the brain, but they were not imaging manifestations of cerebral infarction. Positive serum syphilis spirochete-specific antibodies and rapid plasma reaction test. The same results were subsequently obtained in cerebrospinal fluid tests. Ultimately, the diagnosis of neurosyphilis was established.

**Intervention:**

The patient was treated with oral antiplatelet aggregating drugs and penicillin G intravenously.

**Outcome:**

The patient had progressive exacerbation of limb weakness and grade 1 muscle strength prior to penicillin therapy. After being treated with penicillin, the patient’s muscle strength gradually improved. After 10 days of penicillin treatment, the muscle strength was completely normalized. Follow-up after 6 months suggested good recovery.

**Lessons:**

Neurosyphilis can present as a stroke-like episode. However, in this case, the patient presented with a progressive stroke, but the head MRI showed diffuse lesions. This suggests that we have more head imaging changes in patients with neurosyphilis and are wary of misdiagnosis in the clinical setting.

## 1. Introduction

Neurosyphilis is caused by infection of the central nervous system by the syphilis spirochete. Neurosyphilis can occur at any time after the initial infection, especially in immunocompromised patients.^[1]^

Early neurosyphilis may be asymptomatic and diagnosed only by laboratory tests, or may present with symptoms such as headaches, meningeal spasms, cerebral nerve paralysis, blindness or deafness, while advanced neurosyphilis may present with paralyzing dementia and spinal tuberculosis.^[2]^

Diagnosis of neurosyphilis remains a challenge because neurosyphilis often presents atypically and is often difficult to distinguish from other diseases. The prognosis can be very good, with almost complete recovery if appropriate treatment is started early.

This article reports a case of a patient with neurosyphilis presenting with progressive stroke but no stroke-like changes on head imaging, who had a good prognosis after treatment with deworming.

## 2. Case report

**Figure 1.**
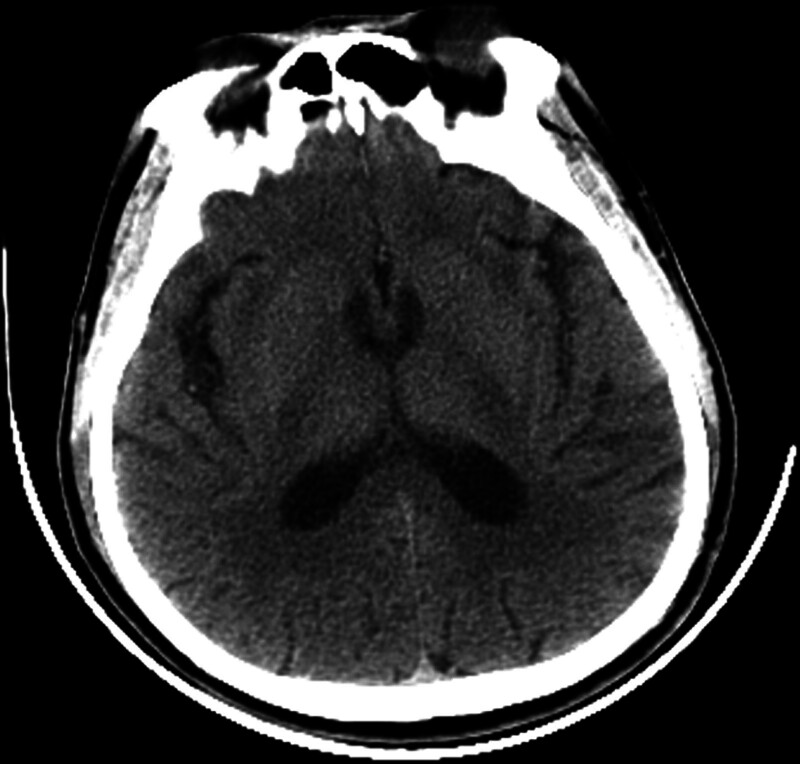
A CT scan of the head on the second day after the onset of the disease showed no abnormal signals. CT = computed tomography.

A 50-year-old male patient with sudden onset of left-sided limb weakness. He arrived at the hospital 1 day after the onset of the illness. The patient had no specific past medical history. History of food and drug allergies is denied. We immediately gave the patient a computed tomography (CT) of the head (Fig. 1), but no abnormal findings were found.

Physical examination: mental clarity, shallow nasolabial groove on the left side, muscle strength grade 4 on the left limb, normal muscle strength on the right limb. Bilateral negative pathologic signs.

**Figure 2.**
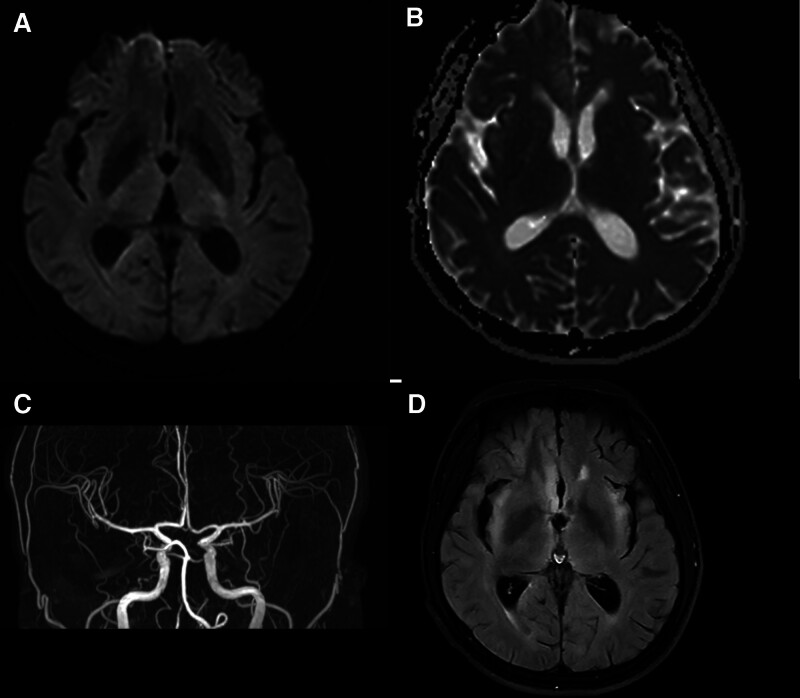
Head MRI findings on the third day of illness. The patient’s head DWI (A) and ADC (B) results were not abnormal and did not support the diagnosis of acute ischemic stroke. MRA of the head showed no intracranial vascular stenosis or occlusion (C). Multiple flaky abnormal signal images are seen on T2 Flair in the bilateral insula, hippocampal region, and lateral paraventricular (D). ADC = apparent diffusion coefficient, DWI = diffusion-weighted imaging, MRI = magnetic resource imaging.

On the third day of illness, the patient underwent an magnetic resource imaging (MRI) of the head, which suggested multiple intracranial abnormal signal images (Fig. 2D), but no abnormality was seen on diffusion-weighted imaging (DWI; Fig. 2A) and apparent diffusion coefficient (Fig. 2B). No vascular stenosis was seen on MRA. Signs were negative (Fig. 2C). However, in the patient’s blood tests, hepatitis C virus antibodies, syphilis spirochete-specific antibodies, and a rapid plasma reaction test were positive. Weakly positive on subsequent plasma reaction 1:8 dilution test.

**Figure 3.**
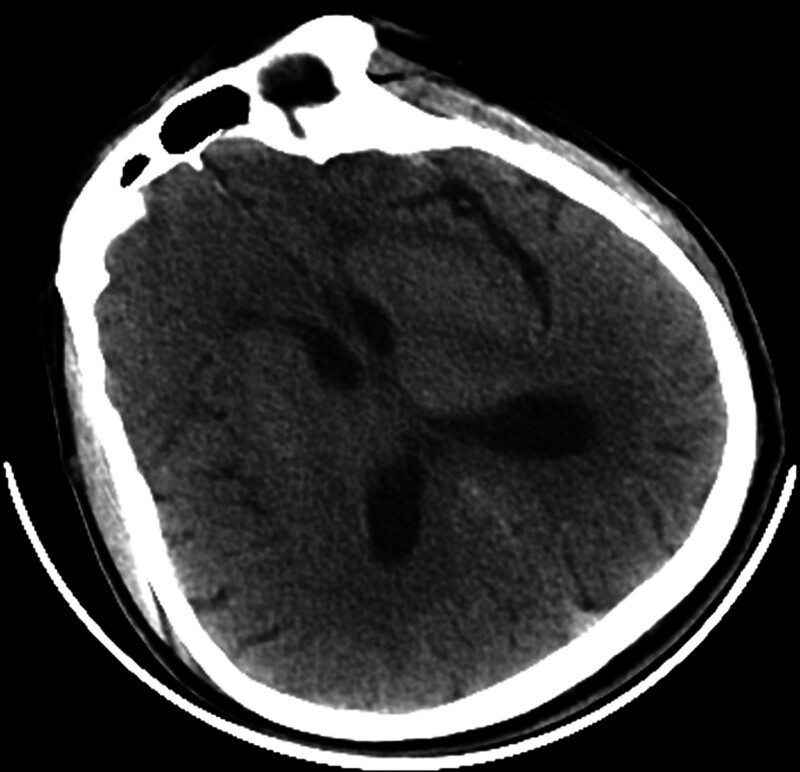
A CT scan of the head on the fifth day of illness still showed no abnormal findings. CT = computed tomography.

On the fourth day of illness, a lumbar puncture was performed. Cerebrospinal fluid pressure was normal. The results are shown in Figure 3.

On the fifth day of illness, the patient’s left limb weakness increased significantly. The muscle strength of the left upper limb was grade 3 and that of the left lower limb was grade 1. The left pupil was 3 mm in diameter and the right pupil was 4 mm in diameter. The patient had severe slurred speech and dysphagia, so a gastric tube was inserted. Pathologic signs on the left side were positive. Reexamine the medical history, the patient presented with decreased arithmetic and unresponsiveness over the past year.

The patient was considered to have neurosyphilis and was treated with penicillin sodium 24 million units daily intravenously. Oral prednisone was also given to prevent Hirschsprung’s reaction.

After being treated with penicillin, the patient’s symptoms improved significantly. On the tenth day of treatment, the patient’s neurological deficit was completely recovered, with normal swallowing function, normal muscle strength of the left limb, and negative pathology on the left side.

**Figure 4.**
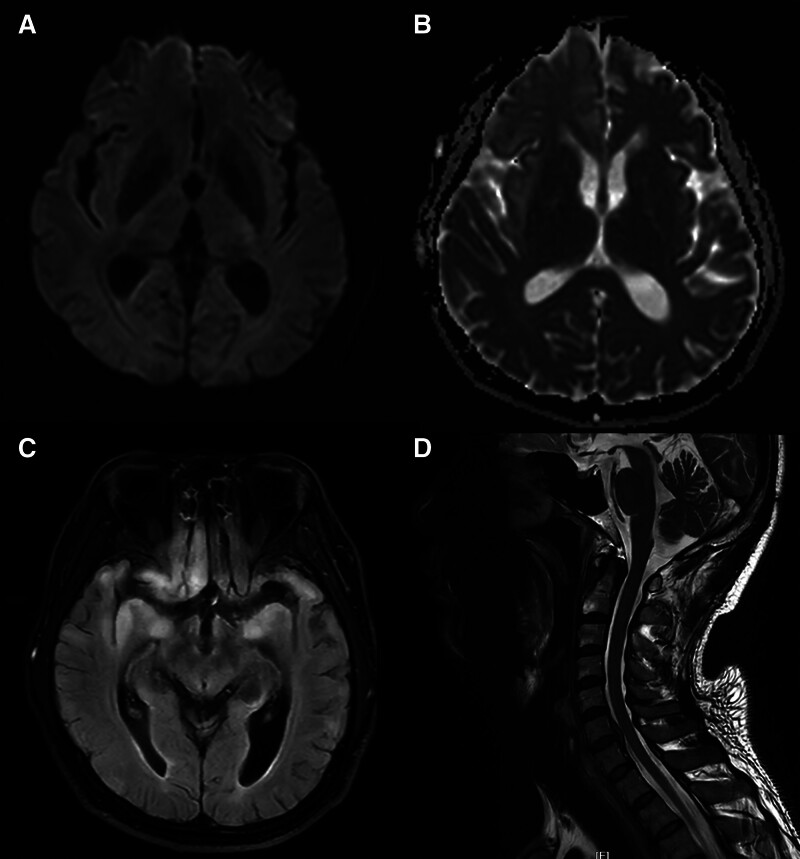
Head MRI findings on the thirteenth day of illness. The patient’s head DWI (A) and ADC (B) results were not abnormal. There is an increase in the number of flaky abnormal signal shadows seen on the T2 flair (C). MRI images of the cervical spine showed no abnormalities (D). ADC = apparent diffusion coefficient, DWI = diffusion-weighted imaging, MRI = magnetic resource imaging.

A head-enhanced MRI examination suggested an increase in intracranial abnormal signal shadows compared with the previous examination (Fig. 4C), but the DWI and apparent diffusion coefficient (Fig. 4A, B) examination result was still negative, and no obvious abnormal enhancement foci were seen in the brain after enhancement. MRI of the cervical spine showed no abnormalities (Fig. 4D).

**Figure 5.**
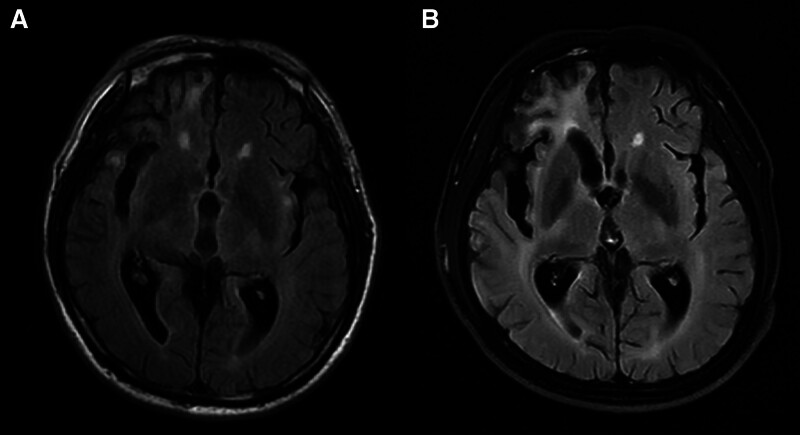
Review of head MRI imaging results after 3 mo of treatment showed a reduction in the patient’s intracranial sheet-like abnormal signal shadow. MRI = magnetic resource imaging.

The results of the MRI of the head were reviewed after 3 months (Fig. 5). The patient had a good prognosis, with complete recovery of limb weakness, but poor memory and numeracy.

## 3. Discussion

Humans are the only host of Treponema pallidum and neurosyphilis is caused by Treponema pallidum invading the central nervous system of the human body, which can be transmitted to the central nervous system at any stage of syphilis infection, rather than the central nervous system as stated in the traditional definition of Treponema pallidum after stage 3 of syphilis. Neurosyphilis occurs in about 4% to 10% of patients with untreated syphilis.^[3]^

Neurosyphilis is an infection of the central nervous system caused by the syphilis spirochete, which can occur at any stage of syphilis infection and is not limited to advanced syphilis. In this case, the patient presented with limb weakness and swallowing dysfunction, symptoms consistent with parenchymal syphilis. In addition, the patient demonstrated A-Ro (Argyll Robertson) pupils, a specific sign of neurosyphilis. At the same time, the patient had experienced a decline in calculation and memory over the past year, and was considered to have paralytic dementia, which is also a characteristic sign of neurosyphilis.

Laboratory tests showed that the patient’s serology was positive for TPPA with a high RPR titer, and the cerebrospinal fluid examination also showed a positive VDRL, findings that further supported the diagnosis of neurosyphilis. Elevated White blood cell count and protein levels in the cerebrospinal fluid are also common manifestations of neurosyphilis.

Paralytic dementia is a late stage manifestation of neurosyphilis, usually presenting years to decades after syphilis infection of the central nervous system, and is usually clinically manifested by personality and behavioral changes (e.g., irritability, emotional lability), cognitive deficits, psychiatric symptoms (e.g., depression, mania, paranoia, hallucinations), delirium, seizures, and sleep disturbances.^[4]^ The most common MRI feature is cortical atrophy, which usually manifests as varying degrees of atrophy and high signal in the frontal lobe, temporoparietal lobe, hippocampus, and corpus callosum on T2WI and magnetic resonance imaging fluid attenuated inversion recovery sequences.^[5]^

In this case, the patient’s head MRI findings showed bilateral temporal lobe and frontal lobe signal abnormalities and subcortical cerebral atrophy consistent with the imaging features of paralytic dementia.

At the same time, the patient had sudden onset of left-sided limb weakness and swallowing dysfunction, which is a manifestation of syphilitic cerebral vasculitis. Unusually, in other patients with neurosyphilis who report stroke-like episodes, head MRI usually has stroke-like changes. This patient presented with stroke-like changes of left-sided limb weakness, left-sided facial and tongue paralysis, and positive left-sided pathology, but the patient’s head CT and 3 head MRIs did not show typical stroke-like imaging changes, which suggests the versatility of our neurosyphilis imaging.

In some patients with non-disabling cerebral infarction or after intravenous thrombolysis, there are usually some patients with negative DWI. These patients usually have mild stroke-like symptoms, but the DWI of the head is negative and no infarct lesions can be seen.

Possible reasons for the clinical occurrence of DWI-negative cerebral infarction include the following: time factor, cytotoxic edema has not yet fully formed in the hyperacute phase (<2 hours), and the degree of water molecule diffusion restriction is insufficient, and the DWI may not be visualized. In the subacute or chronic phase (>7 days), cytotoxic edema subsides and the DWI signal returns to normal. Cavernous infarcts or small cortical infarcts may not be clearly demonstrated by DWI due to partial volume effect or resolution limitations. Venous infarcts, infarcts caused by cerebral venous thrombosis are dominated by vasogenic edema (extracellular edema), and DWI may be negative or mixed signal. Inflammatory and infectious vascular lesions, such as neurosyphilis and vasculitis, are dominated by vasogenic edema due to increased vascular permeability caused by chronic inflammation (DWI-negative).

This patient presented with severe neurological deficits, but the DWI was negative and the symptoms recovered rapidly after penicillin treatment. The possible reason for this is the characteristic change in DWI signal due to differences in pathological mechanisms. Cerebral infarction in neurosyphilis is most often caused by syphilitic vasculitis, which manifests as intimal hyperplasia, inflammatory infiltration and occlusion of small and medium-sized arteries. Unlike atherosclerotic infarction, this chronic inflammation may trigger incomplete ischemia (e.g., hypoperfusion rather than complete occlusion), resulting in less severe impairment of cellular energy metabolism and less pronounced cytotoxic edema, and thus the DWI signal may not be elevated (negative).

However, treatment of neurosyphilis does not only target symptomatic relief, but also requires close monitoring of the patient’s serological and cerebrospinal fluid markers to assess treatment efficacy and disease activity. Patients need to have serological reviews every 3 months and cerebrospinal fluid tests every 6 months for the first year after treatment to ensure that the disease is under control.

In conclusion, this case highlights the diversity and complexity of neurosyphilis and the importance of early recognition and treatment. In patients with atypical clinical presentation, the possibility of neurosyphilis should be highly suspected and appropriate laboratory and imaging tests should be performed. In addition, long-term follow-up and management of patients with neurosyphilis is equally important to prevent recurrence and progression of the disease.

## Author contributions

**Conceptualization:** Ling Zeng.

**Supervision:** Bin Wu.

**Project administration:** Yushi Zhong.

**Writing – review & editing:** Yushi Zhong.
